# Recombinant proteins in plants

**DOI:** 10.1186/1753-6561-8-S4-O1

**Published:** 2014-10-01

**Authors:** Elibio Rech, Giovanni Vianna, Andre Murad, Nicolau Cunha, Cristiano Lacorte, Ana Araujo, Marcelo Brigido, Waters Michael, Aparecida Fontes, O'Keefe Barry, Simpson Andrew, Caballero Otavia

**Affiliations:** 1 Embrapa Genetic Resources and Biotechnology, Parque Estação Biológica, PqEB, Av. W5 Norte, Brasília, DF, 70770-917, Brazil; 2 Departamento of Celular Biology, University of Brasília, Brasília, DF, 70910-900, Brazil; 3 Institute for Molecular Bioscience and School of Biomedical Sciences, University of Queensland, St Lucia, Queensland, Australia; 4 Blood Center of Ribeirão Preto, Center of Celular Therapy - FMRP/USP, Rua Tenente Catão Roxo 2501, Monte Alegre, Ribeirão Preto, SP, 14051-140, Brazil; 5 Molecular Targets Development Program, National Cancer Institute at Frederick, Frederick, MD 21702, USA; 6 Ludwig Institute for Cancer Research Ltd, New York Branch of Human Cancer Immunology at Memorial Sloan-Kettering Cancer Center 1275 York Avenue, BOX 32, New York, NY, 10065, USA

## Background

Over the last few decades, several studies have shown that plants can be a viable option for producing functional recombinant proteins with a wide range of structural characteristics [1]. In addition, the potential benefit for developing countries is a prominent feature that we have recently addressed [2]. Plant-produced recombinant proteins can already be considered a novel component in sustainable food production [3]. A major reason for this optimism relates to cost. Indeed, it is widely recognized that plants used as bioreactors to produce recombinant proteins would enable a significant reduction in overall manufacturing costs [2]. Although recombinant proteins can be functionally expressed in different plant systems, it is imperative to determine the platform that offers the most advantageous conditions for the expression and recovery of a particular protein [1]. In addition, because plant pathogenic organisms cannot cause human disease, this opens the possibility of exploiting plants and edible fruits as potential candidates for the production of orally administered antigens [1]. Basically, there are three strategies for recombinant protein production in plant-based systems: (1) use of cell-culture-based systems that are equivalent to mammalian, microbial and insect cell systems; (2) transient expression of foreign genes in plant tissues that are transformed by either agroinjection or by viral infection and (3) development of transgenic plants carrying stably integrated transgenes [4,5]. Here, I will focus on some of our recent results on transient expression and soybean seed as bioreactor-based systems. Transient expression systems are very useful for research and are now being routinely used for the rapid production of valuable proteins. These systems allow high throughput production and straightforward manipulation, permitting the rapid validation of expression constructs and the production of large amounts of recombinant protein within a few weeks. As a direct consequence, the protein yields from transient expression in plants are normally higher than yields observed in other recombinant plant systems. Transient technology is based on the insertion of transgenes into plant cells using plant viruses, commonly the tobacco mosaic virus (TMV) and the potato virus × (PVX) as well as transgenic *Agrobacterium tumefaciens*. Transgene insertion occurs without stable chromosomal integration, resulting in non-permanent and non-inheritable gene expression. Because the transfer rates of *Agrobacterium *T-DNA and viral-carried genes can reach a very high number of plant cells after infection. Tobacco leaves are the dominant choice for the development of commercial platforms using transient expression [1]. Seeds as bioreactors also provide a potential economical platform for the large-scale production and storage of recombinant proteins [1,5]. Soybean seed storage proteins are of great interest for the development of regulated tissue-specific genes products of commercial interest through recombinant DNA technology. The 7S globulins are comprised of β-conglycinin subunits. β-conglycinin regulatory sequences are seed tissue-specific, temporally regulated and expressed in both the embryonic axis and cotyledons of developing seeds.

## Methods

For transient expression, different constructs were tested containing signal peptides for endosplasmic reticulum (ER), apoplast and chloroplast. To generate transgenic soybean lines, coding sequences of the *human growth hormone; human coagulation factor IX, NY-ESO-1 and cyanovirin *(CV-N) genes were placed under control of different seed-specific promoters and signal peptides. Utilizing biolistic-mediated transformation (Rech *et al*., 2008), each construct was introduced into the soybean genome and putative transgenic plants were obtained [5]. The utilization of a nano-UPLC fractionation and SynaptHigh Definition Mass Spectrometry (HDMS) allowed us to develop a nanoESI positive mode system to conduct a characterization of the recombinant proteins in a single seed with Identity [6].

## Results and conclusions

Transient expression of cancer/testis NY-ESO-1 antigen was detected in *Nicotiana benthamiana *leaves using a PVX-based transient assay. Accumulation in leaf tissue was influenced by the subcellular localization. No signal was detected when the NY-ESO-1 antigen was targeted to chloroplast or to the apoplast, and detection was only possible when the protein was targeted to the ER. NanoUPLC-MS^E ^analysis confirmed the presence of heterologous NY-ESO-1 at a level of approximately 0.1% of the total soluble protein extract (TSP). Exploiting the utilization of seed-specific regulatory sequences was possible to direct and accumulate recombinant proteins in the PSV's from soybean seeds [5]. Expression levels of bioactive human growth hormone (hGH) and human coagulation factor IX (and hFIX) were respectively of up to 2.9% TSP content (corresponding to approximately 9 g kg^-1^) and up to 0.23% (0.8 g Kg^-1^). NY-ESO-1 antigen presented low expression level in soybean seeds (<0,1% TSP). The somatogenic activity bioassay demonstrated that the hGH expressed in soybean seeds is fully active. Protein extracts from transgenic seeds containing the hFIX showed a blood-clotting activity of up to 1.4% of normal plasma. Anti-HIV assays from semi-purified SOY-CV-N, indicated an effective anti-HIV activity in the viral assay. Purification of pure recombinant CV-N in soybean seeds is still ongoing, showing a high affinity for CV-N to bind to glycinin and B-conglycinin proteins from seeds. NanoUPLC-MS^e ^analytical results indicated expression level and correct structural characterization of recombinant protein sequences. Ultrastructural immunocytochemistry assays revealed that the recombinant proteins accumulated in the endoplasmic reticulum (ER)-derived protein storage vacuoles. Recombinant molecules were visualized all along the cistern lumen but were absent from the interior of seed oil bodies. Currently, we are concentrating efforts on studies and development of technologies for genome editing and engineering using TALEN, CRISPR, synthetic chromosomes and super-enhancers [7-9]. This should contribute to: a) understanding the minimum number of bioparts necessary for metabolic function and regulation of gene expression; b) developing synthetic chromosomes, which may enable the accurate expression of gene families; c) developing regulatory circuits and designing synthetic genes that can be based on standard biological parts, as models for the development of synthetic biology tools for engineering genomes. In addition, making use of genomics as a template founded on molecular synthesis and assembly, using recombinant DNA technology and synthetic biology, has allowed the sustainable prospection and manipulation of innovative traits found in biodiversity [10]. The aim includes improvement and development of novel tools for production of recombinant proteins in plant systems.
